# Case Report: Complement-mediated thrombotic microangiopathy masquerading as a pancreatic mass

**DOI:** 10.3389/fmed.2026.1766086

**Published:** 2026-05-07

**Authors:** Chuanchuan Sun, Ying Ding, Fan Yang, Yunfei Ding, Zhen Qu, Feng Yu

**Affiliations:** 1 Department of Nephrology, Peking University International Hospital, Beijing, China; 2 Public Health and Preventive Medicine of Peking University School of Public Health, Peking University, Beijing, China; 3 National Institute of Health Data Science, Peking University, Beijing, China; 4 Department of Nephrology, The Second Medical Center of PLA General Hospital, National Clinical Research Center for Geriatric Diseases, Beijing, China; 5 Department of Nephrology, Beijing Aerospace General Hospital, Beijing, China

**Keywords:** complement-mediated, eculizumab, pancreatic mass, renal injury, thrombotic microangiopathy

## Abstract

**Background:**

Complement-mediated thrombotic microangiopathy (CM-TMA) results from dysregulated alternative pathway activation. Although extra-renal manifestations are well-recognized, presentation as a pancreatic mass is exceedingly rare and risks misdiagnosis as malignancy, delaying critical intervention.

**Case presentation:**

A 28-year-old male presented with malignant hypertension and rapidly progressive renal impairment. Abdominal CT revealed a pancreatic tail mass suspicious for neoplasia. However, histopathology of both renal and pancreatic tissues demonstrated microvascular thrombosis and endothelial injury, pointing to a systemic TMA process. Subsequent genetic testing confirmed pathogenic variants in CFH (p.Tyr1058His, p.Val1060Leu) and THBD (p.Asp486Tyr), establishing the diagnosis of CM-TMA.

**Intervention:**

Treatment was initiated with eculizumab alongside intensive renin-angiotensin system blockade.

**Outcomes:**

After 6 months of therapy, renal function recovered and the pancreatic mass completely resolved. However, 3 months after self-discontinuing eculizumab, the patient experienced a severe disease relapse and subsequently entered maintenance hemodialysis.

**Conclusion:**

This first histopathologically confirmed case of adult CM-TMA presenting as a pancreatic mass expands the known phenotype of complement-mediated disease. It highlights that in young adults with unexplained malignant hypertension and multisystem involvement, prompt evaluation for complement abnormalities is critical. The presence of high-risk complement variants and the relapse following treatment cessation underscore the necessity for long-term complement inhibition in such patients.

## Introduction

Thrombotic microangiopathy (TMA) comprises a group of clinicopathological syndromes characterized by microangiopathic hemolytic anemia, thrombocytopenia, and end-organ damage (1). Complement-mediated TMA (CM-TMA), also referred to as atypical hemolytic uremic syndrome (aHUS) (2, 3), results from dysregulated activation of the alternative complement pathway (1, 4). In recent years, the nomenclature has evolved, with “CM-TMA” now encompassing many cases historically labeled as aHUS, reflecting a broader understanding of complement-driven pathophysiology beyond the classic triad of hemolytic anemia, thrombocytopenia, and renal impairment (3, 5). Although the kidneys are the most frequently involved organs in CM-TMA, extra-renal manifestations affecting the central nervous, cardiovascular, and gastrointestinal systems are not uncommon (6, 7). Although pancreatic involvement in CM-TMA is rare and typically manifests as acute pancreatitis or diabetes mellitus (8–10), a mass-forming pancreatic lesion documented by imaging and confirmed by histopathology in an adult has not, to our knowledge, been previously reported (6, 7, 11). Such atypical presentations are highly susceptible to misdiagnosis as pancreatic tumors, potentially leading to incorrect therapeutic decisions. This report describes a case of CM-TMA presenting with malignant hypertension, acute kidney injury, and a pancreatic mass, ultimately diagnosed through histopathological examination of both renal and pancreatic tissues and genetic sequencing. This case aims to enhance clinicians’ awareness of the rare manifestations of CM-TMA to prevent misdiagnosis and to underscore the efficacy of complement inhibitory therapy.

## Case report

### Initial presentation

A 28-year-old Chinese male was found to have hypertension during a routine physical examination 20 months prior to the current admission and remained untreated. Sixteen months prior to admission, he presented to a local hospital with acute blurred vision. His blood pressure was markedly elevated at 220/160 mmHg, with a serum creatinine (Scr) of 149 μmol/L (estimated glomerular filtration rate [eGFR] 58 mL/min/1.73 m^2^). Funduscopy revealed retinal exudates, leading to a diagnosis of malignant hypertension (MHT). Antihypertensive therapy with amlodipine and carvedilol was initiated.

**Figure 1:**
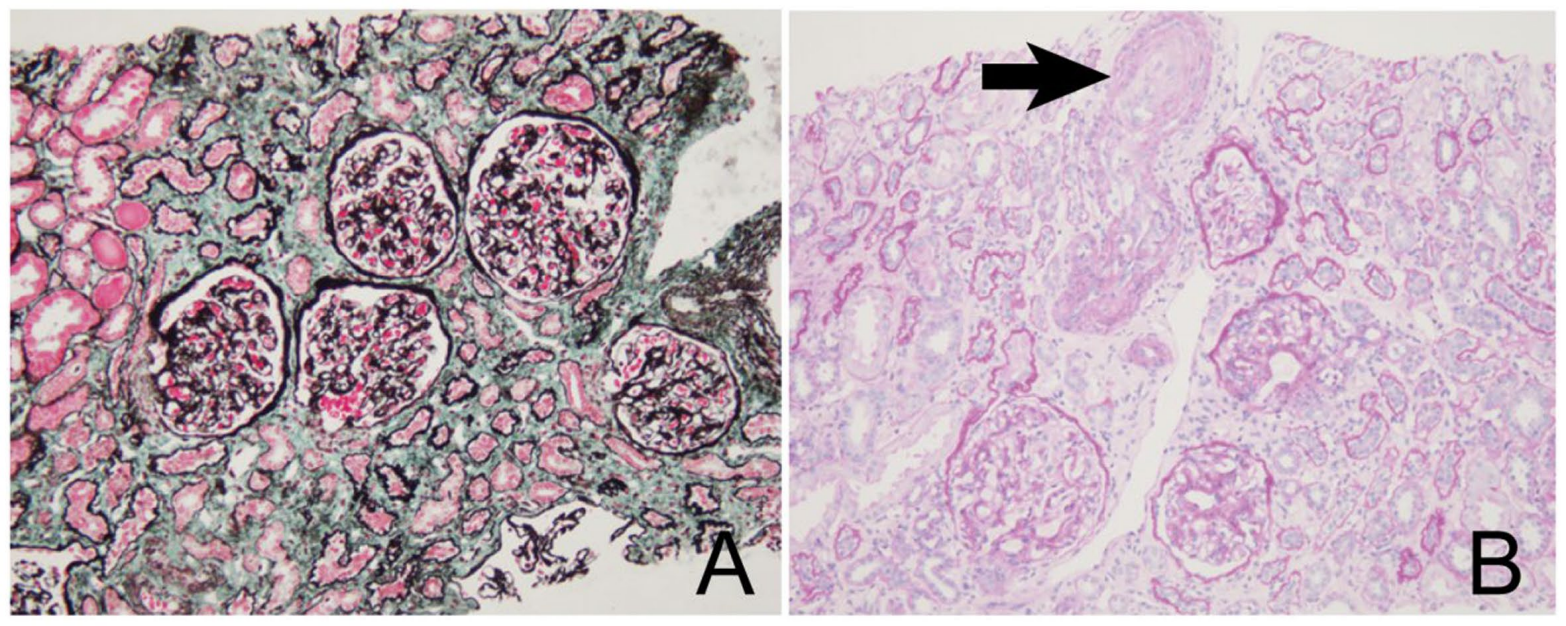
A biopsy of the kidney. **(A)** Showed glomerulus with capillary loops were obviously shrunk and poorly opened, the epithelial cells in the parietal layer were extremely proliferated, and the wall segments of Bowman’s capsule were thickened and stratified (Masson trichrome staining, ×100). **(B)** Showed renal artery intima wall thickening (intimal thickness>media thickness), the arterial wall was thickened, and some arterioles showed mucilaginous changes, endothelial cell foam like changes, lumen stenosis and occlusion (>50%) (arrow head).(periodic acid–Schiff (PAS) staining, ×100).

Due to poorly controlled hypertension, the patient was transferred to a tertiary hospital. Normal renal artery and adrenal Doppler ultrasound excluded secondary hypertension due to renal artery stenosis and adrenal space-occupying lesions. Urinalysis showed proteinuria (++) without microhematuria, with a 24-h urinary protein excretion of 1.9 g. Scr increased to 250 μmol/L (eGFR 55 mL/min/1.73 m^2^). Complement studies showed decreased C3 at 0.75 g/L (reference range 0.9–1.8 g/L) with a normal C4 level. Autoantibody screens (including ANA, ANCA, anti-GBM) and malignancy workup were negative (Tables 1, 2). A renal biopsy was performed, and the pathology was reported as “hypertensive nephropathy” (Figure 1). The antihypertensive regimen was intensified to include nifedipine controlled-release tablets, metoprolol succinate, and irbesartan.

**Table 1:** The baseline laboratory data of the patient.

Parameters		Result	Reference range
Urinalysis	Proteinuria	2+	negative
	Hematuria	negative	negative
	UTP	1.7 g/24 h (1.9 L)	<0.15 g/24 h
Serum comprehensive metabolic panel	Creatinine	203 μmol/L	59-104 μmol/L
	eGFR	37.32 mL/min.1.73m^2^	/
	Albumin	41.2 g/L	40-50 g/L
	Potassium	5.32 mmol/L	3.5–5.3 mmol/L
	TBil	4.8 μmol/L	3.4–23.3 μmol/L
	LDH	134 U/L	120-250 U/L
Immunoglobulin	IgG	10.14 g/L	7.0–16.0 g/L
	IgA	1.21 g/L	0.7–4.0 g/L
	IgM	0.64 g/L	0.4–2.0 g/L
	IgE	37 IU/mL	≤100 IU/mL
Complement	C3	1.19 g/L	0.9–1.8 g/L
	C4	0.27 g/L	0.1–0.4 g/L
	CH50	52.39 U/mL	26-54 U/mL
	Factor B	329 mg/L	100-400 mg/L

eGFR, estimated glomerular filtration rate; LDH, lactate dehydrogenase; TBil, total bilirubin; UTP, urinary total protein.

**Table 2:** The laboratory investigations for secondary etiologies.

Parameters		Result	Reference range
ADAMTS13		72%	/
ANCA assemble	pANCA	negative	<1:10
	cANCA	1:10 (atypical cANCA)	<1:10
	MPO-ANCA	0.6CU	<20CU
	PR3-ANCA	0.8CU	<20CU
	GBM	0.8CU	<20CU
Malignancies screening	AFP	2.3 ng/mL	≤7
	CEA	2.7 ng/mL	≤5
	CA199	5.0 U/mL	≤39
	CA724	1.0 U/mL	≤6.9
	NSE	11.3 ng/mL	≤16.3
Infectious disease indicators	HBsAg	0.00C. O. I	<1C. O. I
	HCV-Ab	0.00C. O. I	<1C. O. I
	HIV-Ag/Ab	0.00C. O. I	<1C. O. I
	TP-Ab	0.00C. O. I	<1C. O. I
Secondary hypertension	Aldosterone	110.1 pg./mL	/
	Angiotensin II	44.43 pg./mL	/
	Direct Renin	344.3μIU/mL	/
	ACTH	61.5 pg./mL	/
Others	PLA2R-Ab	<6RU/mL	0-20RU/mL
	ANA	negative	negative
	Serum/urine protein electrophoresis	no paraprotein and a normal free light chain ratio	/

ACTH, adrenocorticotropic hormone; ADAMTS13, a disintegrin and metalloprotease with thrombospondin type 1 motif member 13; AFP, Alpha fetoprotein, ANA, anti-nuclear antibodies, ANCA, anti-neutrophil cytoplasmic antibody; pANCA, perinuclear anti-neutrophil cytoplasmic antibody; cANCA, cytoplasmic anti-neutrophil cytoplasmic antibody; CA199, carbohydrate antigen199; CA724, carbohydrate antigen724; CEA, carcinoembryonic antigen; GBM, glomerular basement membrane; HBsAg, hapatitis B surface antigen; HCV-Ab, hepatitis C virus-antibody; HIV-Ag/Ab, human immunodeficiency virus-antigen/antibody; MPO, myeloperoxidase; NSE, neuron-specific enolase; PR3, proteinase 3; PLA2R-Ab, anti- M-type phospholipase A2 receptor antibody; TP-Ab, *Treponema pallidum*-antibody.

### Development of pancreatic mass

**Figure 2:**
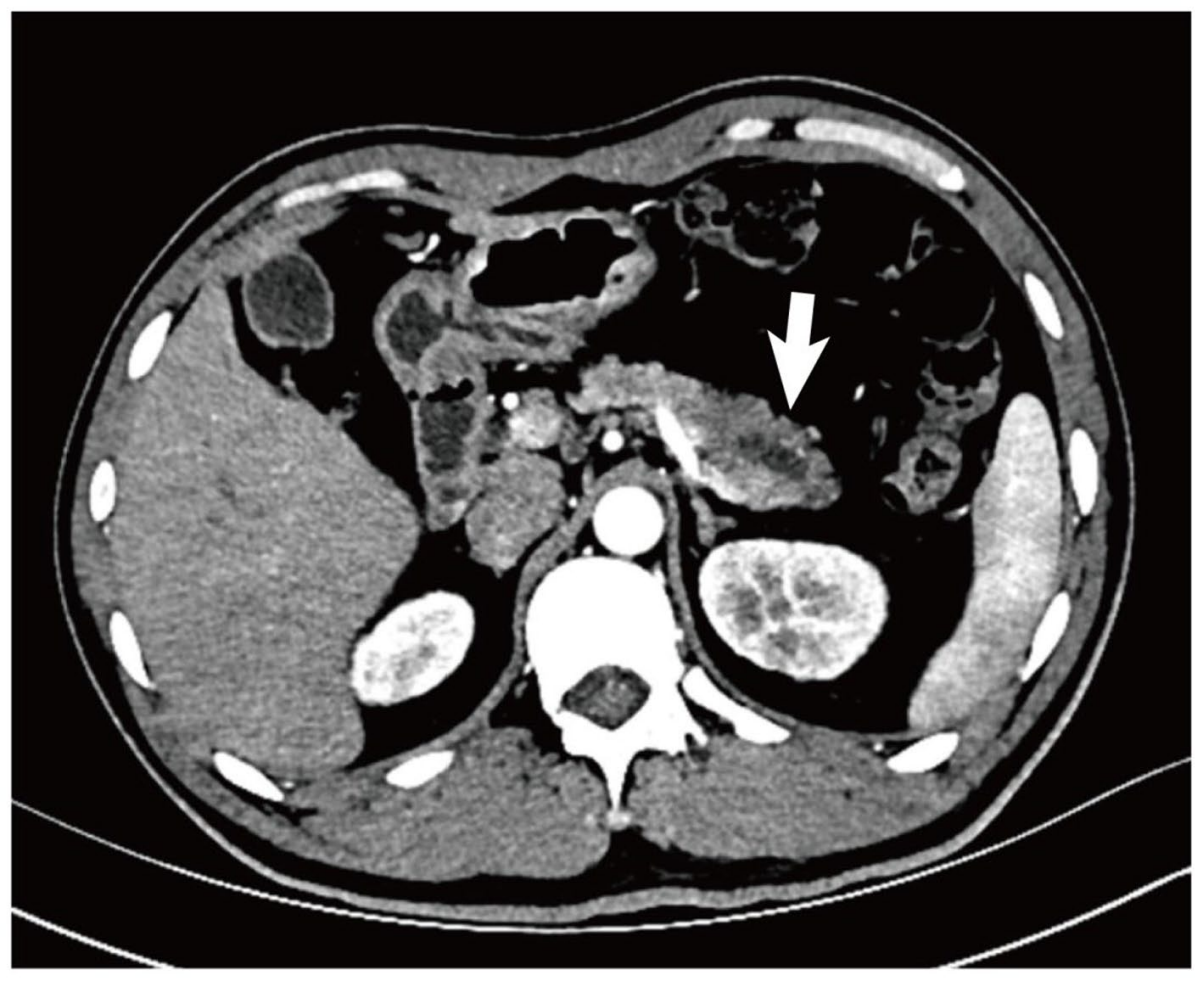
Abdominal enhanced computed tomography (CT) showed thickening of the pancreatic tail with hypodense shadows, suggesting a suspicious pancreatic mass (white arrow head).

**Figure 3:**
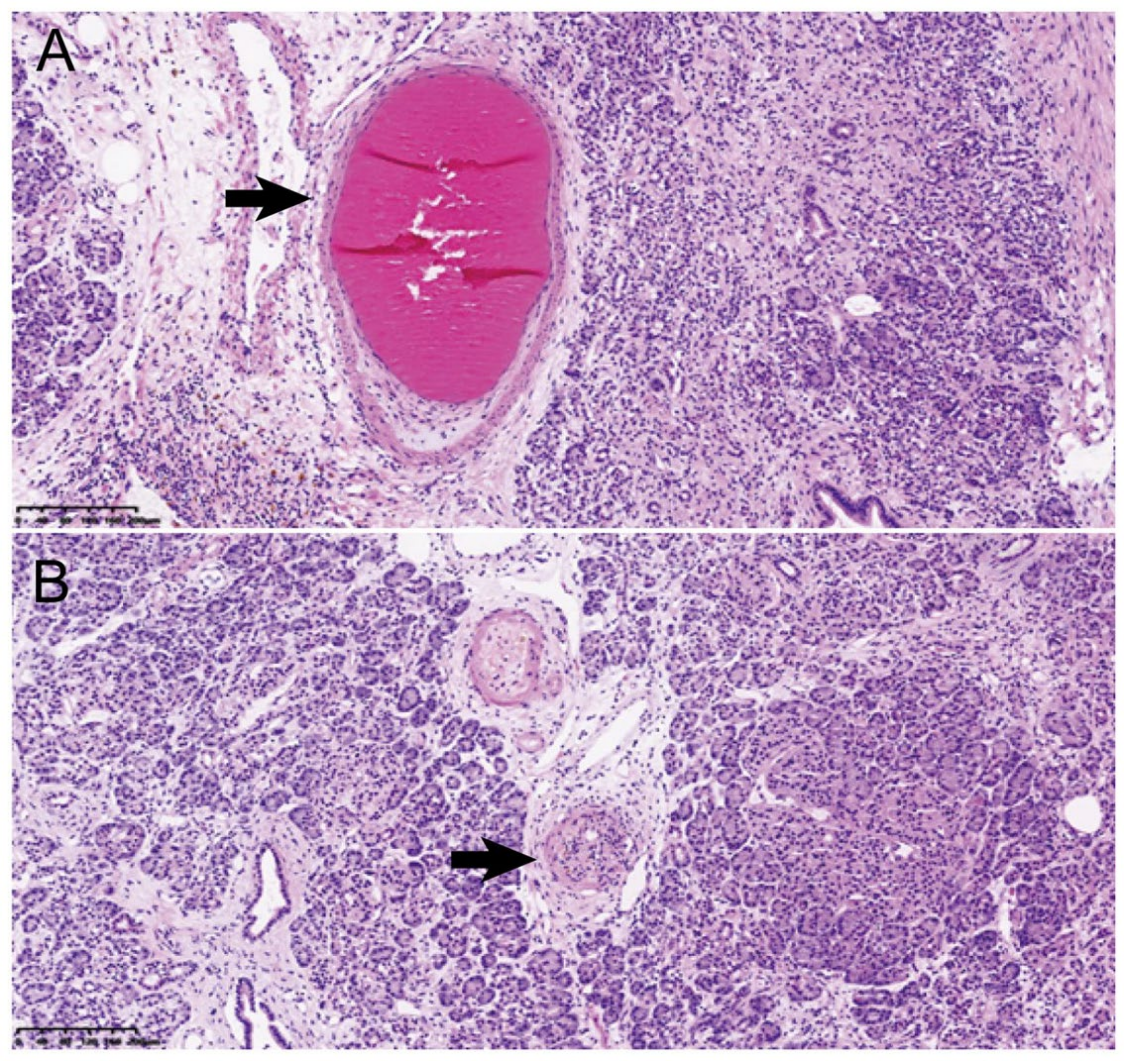
Pathology of resected pancreatic sections. **(A)** Showed the thrombi in the vascular lumen (arrow head). **(B)** Showed hyperplasia and swelling of vascular endothelial cells, and intimal thickening of blood vessels with mucoid changes (arrow head) (Hematoxylin and eosin staining, ×100).

The patient was hospitalized again one month prior to admission due to low back pain. Laboratory tests revealed progressive deterioration of renal function, with Scr rising to 371 μmol/L, and persistently low C3 (0.68 g/L). Abdominal CT at the referring hospital demonstrated thickening of the pancreatic tail, suspicious for a mass lesion (Figure 2). After multidisciplinary evaluation (gastroenterology, pancreatic surgery, radiology, nephrology), distal pancreatectomy was recommended despite normal serum tumor markers (CA19-9: 5.0 U/mL; carcinoembryonic antigen [CEA]: 2.7 ng/mL). Imaging findings were highly suspicious for malignancy, and given the patient’s young age and multisystem involvement (kidneys, ocular fundus, cardiovascular system), the consensus favored a pancreatic neoplasm. Surgery was therefore undertaken to definitively exclude solid tumors, including ductal adenocarcinoma and solid pseudopapillary neoplasm. Postoperative pathological examination showed architectural disruption of the pancreatic tissue, fibrotic replacement, neutrophilic infiltration, and microangiopathic changes characterized by endothelial swelling and intraluminal thrombi (Figure 3). Following surgery, his blood pressure stabilized, and Scr decreased to approximately 200 μmol/L.

### Transfer and definitive diagnosis

He was subsequently transferred to our institution for further management. On admission, vital signs were stable, with a blood pressure of 114/82 mmHg (on multiple antihypertensive agents). Physical examination was unremarkable. Laboratory investigations revealed: Scr 203 μmol/L (eGFR 39 mL/min/1.73 m^2^), hemoglobin 111 g/L, persistently low C3 (0.88 g/L), and normal soluble C5b-9. Genetic test results identified pathogenic heterozygous variants in *CFH* (c.3172T>C, p.Tyr1058His; c.3178G>C, p.Val1060Leu) and *THBD* (c.1456C>A, p.Asp486Tyr) (Table 3).

**Table 3:** Genetic analysis revealed the patient had mutations in complement factor H (c.3172 T>C, c.3178G>C), thrombomodulin (THBD) (c.1456C>A), complement factor H related protein 3 (CFHR3, c.721C>T), complement receptor 1 (CR1, c.3066G>T).

Gene (transcript)	Nucleotide changes	Amino acid changes	Chromosomal location	Thousand person frequency		Heterozygosity	Family variation		Protein function damage	ACMG classification
				1000G	ExAC_ East Asia		Father	Mother		
CFH (NM_000186)	c.3172 T>C	p.Y1058H	chr1: 196712620	0.0014	0.0097	hybrid	-	-	T; B; N; L; T; N;-4.08	VUS
CFH (NM_000186)	c.3178G>C	p.V1060L	chr1: 196712626	-	0.0096	hybrid	-	-	T; B; N; L; T; N; −10.3	VUS
THBD (NM_000361)	c.1456C>A	p.D486Y	chr20: 23028686	0.0052	0.0135	hybrid	-	-	T; P; N; N; T; N;-5.26	VUS
CFHR3 (NM_021023)	c.721C>T	p.P241S	chr1: 196759282	-	0.0117	homozygous	-	-	T; B; P; N; T; N;-6.54	VUS
CR1 (NM_000651)	c.3066G>T	p.Q1022H	chr1: 207726161	-	-	hybrid	-	-	T; D; N; L; T; N;-0.164	VUS*

ACMG, American College of Medical Genetics and Genomics; VUS, Variant of uncertain significance.

The diagnosis of CM-TMA was established based on the following findings: (i) persistent hypocomplementemia with low C3 and normal C4 levels, indicative of alternative complement pathway dysregulation; (ii) identification of heterozygous variants in CFH (p.Tyr1058His and p.Val1060Leu) and THBD (p.Asp486Tyr), which are known to confer susceptibility to CM-TMA; and (iii) histopathological evidence from both renal and pancreatic tissues demonstrating characteristic features of TMA, including microvascular endothelial injury and microthrombi formation. Collectively, these findings supported that the patient’s hypertension, renal impairment, and pancreatic involvement were systemic manifestations of CM-TMA, establishing the final diagnosis of CM-TMA.

The differential diagnosis comprehensively excluded other forms of TMA and secondary etiologies. A normal ADAMTS13 activity level (72%) excluded thrombotic thrombocytopenic purpura (TTP). The absence of prodromal diarrhea or bloody stools, along with negative stool studies and no evidence of infection, ruled out Shiga toxin-producing *Escherichia coli*-associated hemolytic uremic syndrome (STEC-HUS). There was no history of drug exposure or thromboembolic events, and comprehensive screenings for malignancy and autoantibodies—including ANA, ANCA and anti-GBM antibody—were all negative. These findings effectively ruled out drug-induced TMA, malignancy-associated TMA, antiphospholipid syndrome (APS), and autoimmune disease-related TMA (e.g., lupus nephritis, ANCA-associated vasculitis). With respect to secondary hypertension, Doppler ultrasonography of the renal arteries and adrenal glands revealed no abnormalities, and there were no clinical features suggestive of endocrine hypertension, thereby excluding renal artery stenosis, adrenal masses, and endocrine-mediated hypertension. Pathological examination of the resected pancreatic specimen confirmed the absence of primary pancreatic malignancy.

### Treatment and follow up

**Figure 4:**
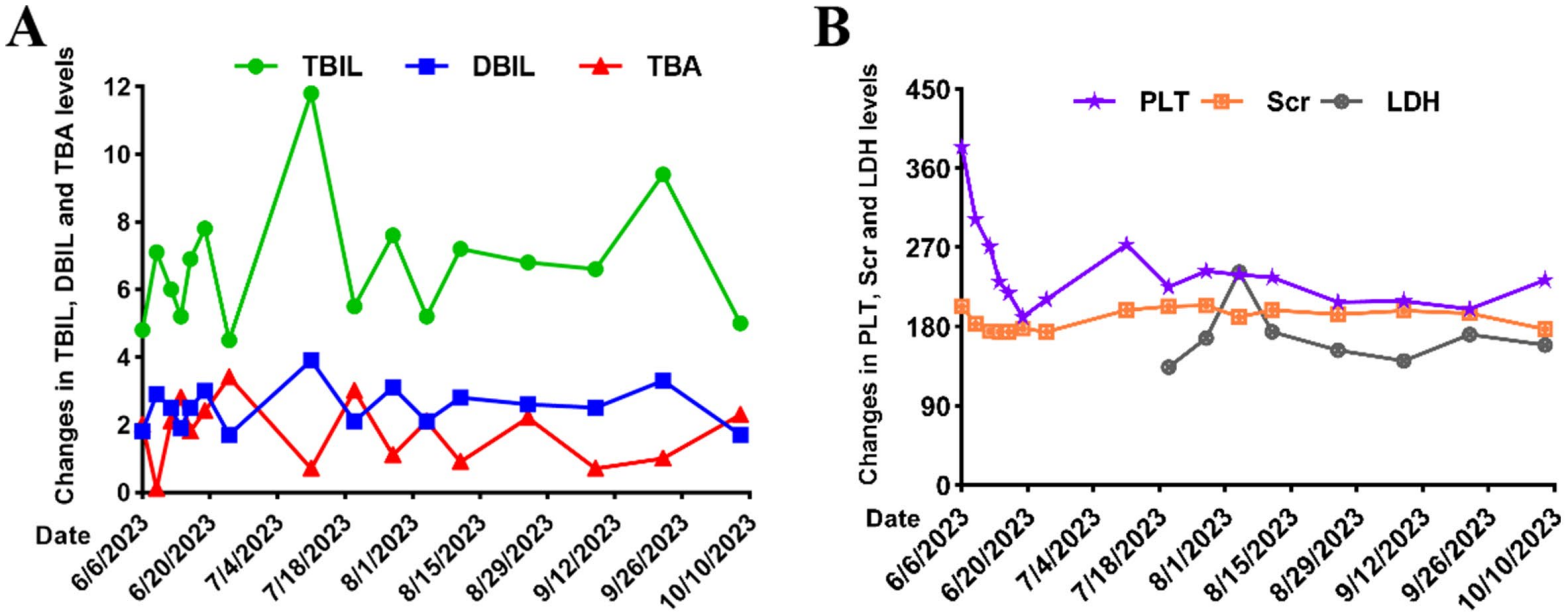
Clinical parameters during hospitalization. **(A)** Serial changes in bilirubin fractions (TBIL, total bilirubin; DBIL, direct bilirubin; TBA, total bile acids; unit, μmol/L). **(B)** Trends in PLT (platelet count, unit: 10^9^/L), Scr (serum creatinine, unit: μmol/L), and LDH (lactate dehydrogenase, unit: U/L) levels.

**Figure 5:**
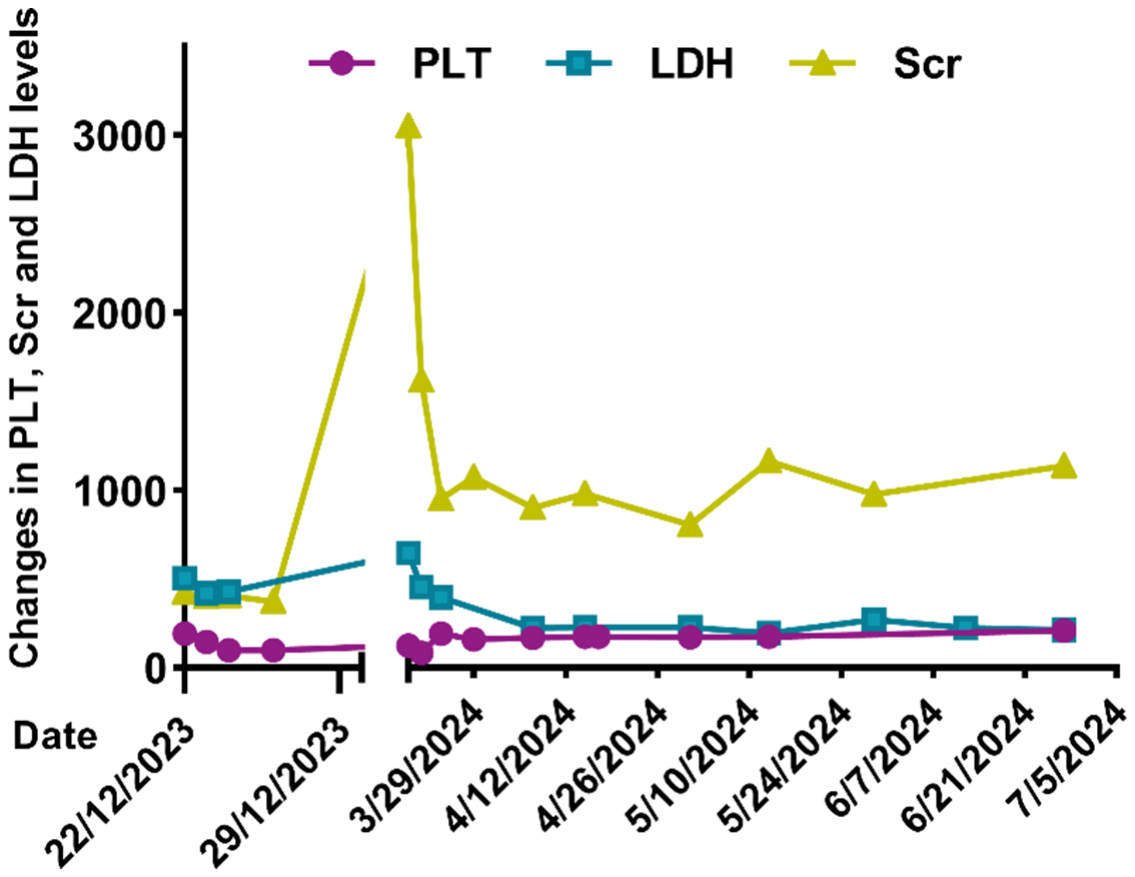
Changes in PLT, LDH, Scr in the patient following discharge from our hospital.

In addition to intensified antihypertensive therapy, eculizumab treatment was initiated (July 14, 2023) approximately 2 weeks after the patient received meningococcal vaccination (June 27, 2023) with an induction regimen of 900 mg administered intravenously weekly for 4 weeks. Followed by a maintenance regimen of 1,200 mg intravenous infusion every 2 weeks. Figure 4 illustrated the serial changes in bilirubin fractions, PLT, Scr, and LDH levels during hospitalization and treatment with eculizumab. After 6 months of eculizumab therapy, the patient’s renal function significantly improved: Scr stabilized at 177 μmol/L (eGFR 44 mL/min/1.73 m^2^), 24-h urinary protein decreased to 0.6 g, and serum albumin normalized (45 g/L). Follow-up abdominal CT showed complete resolution of the pancreatic mass. Complement activity monitoring indicated effective suppression of the classical pathway (CH50 0.93 U/mL). Unfortunately, due to financial constraints, the patient self-discontinued eculizumab. This resulted in disease relapse 3 months later, with serum creatinine rising to 422 μmol/L and refractory hypertension. Figure 5 illustrated the longitudinal changes in Scr, PLT, and LDH levels during follow-up at an outside institution following the patient’s discharge from our hospital. The patient soon required maintenance hemodialysis.

## Discussion

This patient’s course, progressing from malignant hypertension and renal impairment to the discovery of a pancreatic mass-like inflammatory/fibrotic lesion, culminating in a diagnosis of CM-TMA via multi-organ histopathology and genetic analysis, highlights the phenotypic complexity of this disease.

### CM-TMA and pancreatic involvement: from inflammation to “mass”

The exact incidence of pancreatic involvement in CM-TMA is unknown. It most commonly manifests as transient elevation of pancreatic enzymes, with only 1–3% of cases progressing to chronic pancreatitis or diabetes mellitus (6, 12). The most striking feature of this case is the imaging finding of mass-like inflammatory fibrotic process in the pancreas, an occurrence that, to our knowledge, has not been previously documented in CM-TMA. To substantiate the novelty of this case, we performed a systematic literature review using PubMed and Web of Science databases (1982–2025) with the search strategy: (“thrombotic microangiopathy” OR “TMA” OR “hemolytic uremic syndrome” OR “HUS” OR “aHUS”) AND (“pancreas” OR “pancreatic” OR “pancreatitis” OR “diabetes” OR “pancreatic insufficiency”). We explicitly excluded pediatric cases (age <18 years) to focus specifically on adult-onset CM-TMA, as the clinical spectrum and genetic background may differ between children and adults. Cases of Shiga toxin-producing *Escherichia coli*-associated HUS (STEC-HUS) were also excluded, given its distinct pathophysiology from complement-mediated TMA. This review identified acute pancreatitis as the predominant pancreatic manifestation in previously reported TMA cases with pancreatic involvement (Table 4). Notably, no cases of mass-forming pancreatic lesions—whether in adults or children, and regardless of STEC-HUS status—were identified in the literature. This systematic approach confirmed that our case represents the first histopathologically confirmed adult CM-TMA presenting as a pancreatic mass. Microvascular thrombosis and endothelial injury in the pancreas can lead to local tissue ischemia, inflammation, and reparative hyperplasia, which may underlie the formation of a mass-like appearance (1). This phenomenon underscores the importance of considering CM-TMA in the differential diagnosis of an unexplained pancreatic mass, particularly when coexisting with renal injury and difficult-to-control hypertension.

**Table 4:** Systematic review of pancreas-related TMA.

Authors	Year	Sex (age)	Primary disease	Involved organs	Treatment	Outcome
Beaudoin et al. (19)	2025	Female (4)	STEC	Pancreas, kidney	Hemodialysis	EPI
Farrugia et al. (20)	2024	Male (31)	AP	Pancreas, kidney	Medical treatment of AP	Recovery
Livingston et al. (21)	2023	Male (38)	AP	Pancreas, kidney	Eculizumab	PPC
Jean-Marie et al. (22)	2020	Male (32)	Alcohol-induced AP	Kidney	Eculizumab	Recovery
Moulis et al. (23)	2012	Male (49)	AP	Pancreas	Prednisone, PE	Recovery
Moulis et al. (23)	2012	Female (29)	AP	Pancreas	Prednisone, PE, rituximab	Recovered after one relapse
Mas et al. (24)	2007	Male (12)	Abdominal injuries, AP	Pancreas, kidney	PE	PPC
Ashraf et al. (25)	2006	Male (2)	STEC	Pancreas, kidney	Hemodialysis	Atrophic pancreas, EPI
Rebouissoux et al. (26)	2004	Female (3)	AP	Pancreas, kidney	Total parenteral nutrition	PPC, chronic renal failure
Bong et al. (27)	2002	Male (35)	AP	Pancreas, kidney	PE, steroids	Recovery
Burns et al. (28)	1982	Female (9)	Unknown	Pancreas, kidney	Steroids	ESRD, pancreatic insufficiency and calcinosis
Andreoli et al. (29)	1982	Female (3)	STEC	Pancreas, kidney, colon	Peritoneal dialysis	Death

STEC, Shiga toxin-producing *Escherichia coli*; EPI, Exocrine pancreatic insufficiency; AP, Acute pancreatitis; PPC, Pancreatic pseudocyst; ESRD, End-stage renal disease; PE, Plasma exchange.

### Complement dysregulation, malignant hypertension, and genotype

Malignant hypertension serves as both a common trigger and a cardinal clinical manifestation of CM-TMA, with the two entities engaging in a bidirectional interplay that perpetuates a vicious cycle (13). The patient, a young male with low pretest probability of primary hypertension, underwent a comprehensive reassessment to exclude all potential secondary etiologies. Through systematic imaging and laboratory evaluation, common secondary causes—including renovascular hypertension, primary aldosteronism, pheochromocytoma, and Cushing’s syndrome—were rigorously ruled out. Notably, while malignant hypertension per se can precipitate secondary thrombotic microangiopathy, it does not typically engender aberrant complement activation nor does it constitute the genetic basis for complement gene variants. Consequently, we concluded that TMA in this patient represented the primary driver of malignant hypertension rather than a secondary phenomenon. Genetic analysis subsequently identified variants in *CFH* (p.Tyr1058His, p.Val1060Leu) and *THBD* (p.Asp486Tyr). Although the pathogenicity classification of these variants remains subject to debate within the ClinVar and HGMD databases, the presence of multiple complement gene variants in patients with aHUS has been robustly associated with a significantly elevated risk of relapse following treatment discontinuation (14)—a finding that aligns closely with the clinical trajectory observed in this case. Furthermore, functional studies have conclusively demonstrated that these genetic variants result in dysregulated activation and hyperactivation of the alternative complement pathway (15, 16); accordingly, we adjudicated both variants as pathogenic. Complement activation products, such as C5a, directly activate endothelial cells, thereby promoting proinflammatory and procoagulant cascades that exacerbate systemic microvascular injury (6). This pathophysiological insight mechanistically explains the limited therapeutic efficacy of intensive antihypertensive monotherapy in this patient, whereas targeted complement inhibition effectively abrogates disease progression at its pathogenic origin.

### Implications of complement inhibition

Eculizumab, a terminal complement C5 inhibitor, is the standard therapy for CM-TMA (17, 18). The concurrent observation of improved renal function and resolution of the mass-like inflammatory fibrotic process of the pancreas in our case strongly demonstrates the central role of complement activation in multi-organ damage. Disease relapse upon drug discontinuation confirms the chronic nature of CM-TMA and suggests that long-term, potentially lifelong, therapy may be necessary for patients carrying high-risk genetic variants (6, 14). This also highlights the urgent need to address issues of drug accessibility and financial burden in clinical practice.

## Conclusion

This case provides the first histopathological confirmation that CM-TMA can manifest as a pancreatic mass lesion, significantly expanding the recognized clinical spectrum of this disease. A high index of suspicion for CM-TMA is warranted in patients with multisystem involvement, particularly affecting the kidneys and pancreas, accompanied by complement abnormalities. Prompt genetic diagnosis and early, long-term complement inhibitory therapy are crucial for improving clinical outcomes. Moreover, in young patients presenting with malignant hypertension, renal impairment, and atypical pancreatic imaging findings, early complement evaluation and multidisciplinary discussion should be considered before proceeding with major surgical interventions whenever clinically feasible.

## Data Availability

The raw data supporting the conclusions of this article will be made available by the authors, without undue reservation.
